# Sociotechnical approaches to workplace safety: Research needs and opportunities

**DOI:** 10.1080/00140139.2015.1011241

**Published:** 2015-03-02

**Authors:** Michelle M. Robertson, Lawrence J. Hettinger, Patrick E. Waterson, Y. Ian Noy, Marvin J. Dainoff, Nancy G. Leveson, Pascale Carayon, Theodore K. Courtney

**Affiliations:** ^a^ Liberty Mutual Research Institute for Safety, 71 Frankland Road, Hopkinton, MA01748, USA; ^b^ Human Factors and Complex Systems Group, Loughborough University, LeicestershireLE11 3TU, UK; ^c^ Massachusetts Institute of Technology, 77 Massachusetts Avenue, Building 33-334, Cambridge, MA02139, USA; ^d^ University of Wisconsin – Madison, 3126 Engineering Centers Building, 1550 Engineering Dr., Madison, WI53706-1609, USA

**Keywords:** sociotechnical systems, occupational safety, complexity, systems theory

## Abstract

The sociotechnical systems perspective offers intriguing and potentially valuable insights into problems associated with workplace safety. While formal sociotechnical systems thinking originated in the 1950s, its application to the analysis and design of sustainable, safe working environments has not been fully developed. To that end, a Hopkinton Conference was organised to review and summarise the state of knowledge in the area and to identify research priorities. A group of 26 international experts produced collaborative articles for this special issue of *Ergonomics*, and each focused on examining a key conceptual, methodological and/or theoretical issue associated with sociotechnical systems and safety. In this concluding paper, we describe the major conference themes and recommendations. These are organised into six topic areas: (1) Concepts, definitions and frameworks, (2) defining research methodologies, (3) modelling and simulation, (4) communications and decision-making, (5) sociotechnical attributes of safe and unsafe systems and (6) potential future research directions for sociotechnical systems research.

**Practitioner Summary:** Sociotechnical complexity, a characteristic of many contemporary work environments, presents potential safety risks that traditional approaches to workplace safety may not adequately address. In this paper, we summarise the investigations of a group of international researchers into questions associated with the application of sociotechnical systems thinking to improve worker safety.

## 1. Introduction

Workplace safety is a persistent, international concern. Many decades of concerted effort within numerous scientific and technical disciplines have contributed to substantial decreases in worker injuries and fatalities, but the problem is far from solved. Moreover, the increasing sociotechnical complexity of contemporary work environments suggests that previously rare or non-existent forms of risk, such as unforeseen, maladaptive interactions between organisational and/or technical system components, will become more common (Carayon et al. 2015; Dekker 2015; Leveson 2012; Wilson 2014). In response to these trends, a growing number of researchers and practitioners are turning to systems-based approaches to workplace safety, with particular focus on the examination of the interactive influences of social-organisational and technical aspects of the work environment (e.g. Underwood and Waterson 2014; Wilson 2014). In light of these developments, the *Hopkinton Conference on Sociotechnical Systems and Safety* was organised in October, 2012 (Noy et al. 2015). The purpose of the conference was two-fold: (1) To identify the gaps in our current understanding of the structure and dynamics of sociotechnical systems insofar as they impact workplace safety and (2) to identify and define potentially productive research directions and priorities.

This paper summarises the key findings and recommendations as discussed in the papers produced by conference participants and presented in this special issue of *Ergonomics.* The major topic areas addressed are: (1) Concepts, definitions and frameworks, (2) defining research methodologies for sociotechnical systems and safety, (3) modelling and simulation of sociotechnical systems, (4) communications and decision-making within sociotechnical systems, (5) sociotechnical attributes of safe and unsafe systems and (6) potential future research directions for sociotechnical systems research. In addition, the results of a conference-wide discussion of future directions in research on sociotechnical systems and workplace safety are summarised.

## 2. Concepts, definitions and frameworks

In their paper, Carayon et al. (2015) present an overarching case for the application of sociotechnical systems thinking to the study and practice of workplace safety. In so doing, they define and critically discuss key sociotechnical concepts as they relate to this area of concern. They begin by defining a sociotechnical system as ‘the synergistic combination of humans, machines, environments, work activities and organisational structures and processes that comprise a given enterprise’ (p. XX_). Following Mumford (2006), they further define a sociotechnical system as comprising two interrelated sub-systems: (1) A technology sub-system, including tools (hardware, software), techniques and work processes and (2) the social or organisational sub-system, including individuals and teams along with the corresponding structure that enables coordination and control.

Equally fundamental is the authors' conception of occupational safety as an *emergent property* of the dynamic interactions between social and technical components. Specifically, safety is defined as the degree of protection against harm afforded by a particular work system, which emerges as a function of this dynamic pattern of interaction and behaviour. The concept of emergence upon which this definition rests is fundamental to the broader theoretical framework of general systems theory (von Bertalanffy 1968) and more recent theoretical treatments of complex adaptive systems (e.g. Holland 2012; Miller and Page 2007; Reiman et al. 2015) in general. From the sociotechnical perspective, safety cannot accurately be said to be ‘a product of’ or to ‘reside within’ one or more of the social and/or technical components of a work system. Rather its presence or absence is a function of (i.e. emerges from) the interactive properties and activities of its constituent components. Given the frequently dynamic nature of sociotechnical system components and their interactions, safety also cannot be said to be a constant or permanent property of a system (i.e. once having been achieved, is achieved ‘once and for all’). This definition has many important implications for the study and practice of workplace safety, one of which is to de-emphasise traditional areas of concern such as component reliability and worker behaviours (e.g. Leveson 2012). Instead, sociotechnical systems thinking proposes a deliberately holistic approach that addresses integrated social and technical influences on overall system dynamics and emergent system properties such as safety.

Carayon et al. (2015) examine many of the key concepts in sociotechnical systems thinking to establish their theoretical and practical relevance within the context of workplace safety. For example, the concept of ‘joint optimisation’ has been an important concept in sociotechnical systems thinking for many years (e.g. Hendrick and Kleiner 2002; Clegg 2000; Kasvi et al. 2000; Kelly 1978; Klein 2014). Simply put, joint optimisation refers to an (ideally) ongoing process comprising thoughtful, disciplined assessment of potential trade-offs involved in personnel- and technology-related workplace decisions. Many accidents that have occurred after significant reductions in workforce (e.g. the Bhopal disaster) can be partially attributed to an asynchronous evolution (Leveson 2012) of technical and personnel resources, or a failure to strive for their joint optimisation. Asynchronous evolutions of this type are a key characteristic of what has been described as organisational ‘drift into failure’ (Dekker 2011).

Having defined these and other related concepts (e.g. the importance of participatory design, managing trade-offs between ‘acute’ and ‘chronic’ system goals), the authors conclude with a set of recommendations for future research. These include the examination of interactions between hierarchical levels of sociotechnical systems and their impact on safe work performance – a recommendation taken up in the paper by Flach et al. (2015) in their analysis of communications and decision-making as mechanisms for visibility and control across hierarchical levels of such systems. Further recommendations include the study of the impact of socio-organisational context on worker safety and the identification of invariant properties and characteristics of sociotechnical systems that promote safety, a topic addressed in the paper by Kleiner et al. (2015).

## 3. Defining research methodologies for sociotechnical systems and safety

Waterson et al. (2015) discuss methodological issues surrounding the empirical analysis of workplace safety from a sociotechnical perspective. Their objective is to outline a set of requirements for use in guiding the development of future research methods. Six case studies are presented as vehicles for discussion of existing, sociotechnically based research methods, which are then systematically evaluated with regard to their ability to address key theoretical and practical issues in workplace safety. The results of their evaluation reveal a number of gaps in the applicability of the methods under examination, leading to the discussion of a set of new methodological requirements.

The authors approach the topic of research methods in the study of sociotechnical systems and safety by placing the problem of workplace safety within the context of key, historical scientific and technical developments. Taking the analysis of Hale and Hovden (1998) as a starting point, Waterson et al. trace the development of the scientific study of safety through three separate ‘ages’: (1) The period extending from the nineteenth century until the Second World War in which safety was primarily addressed from a technology-centred, interventional perspective; (2) the ‘age of human factors’ when concerns with the potential impact of human performance capabilities and limitations (physical, perceptual, cognitive, etc.) began to be introduced in to existing risk and safety analyses and (3) a contemporary stage in which concern with complex system failures and subsequent accidents and disasters began to generate interest in the examination of underlying sociotechnical factors. This historical overview captures the evolution of work-based systems towards greater sociotechnical complexity, and the corresponding requirement to develop empirical assessment techniques that can adequately account for such complexity.

The six case studies presented by Waterson et al. cover a broad array of applications and techniques, and the reader is encouraged to consult their paper for detailed discussions of each. To summarise, the work domains presented included health care, railroad operations, unmanned aerial system operation, knowledge work and submarine control activities. The methods used in each were equally diverse and included surveys, interviews, observations, cognitive task analyses, a ‘Systems Analysis Tool’ (SAT) technique derived from macroergonomics principles (Robertson and Courtney 2004; Robertson, Kleiner, and O'Neill 2002) and Event Analysis of Systemic Teamwork (EAST), a command and control assessment technique (Stanton, Baber, and Harris 2008).

While Waterson et al. proceed to critically assess each method (as well as others) as deployed within its specific, case study application and with respect to other potential applications, it is clear that each share a number of key attributes. For example, sociotechnically based methods of the type presented are acutely sensitive to *context*; that is, they not only seek to illuminate the immediate organisational and technical factors that impact safe work performance, but also the less immediate and more diffuse influences of organisational policy, communications and decision-making as they ‘flow down’ to influence performance. To adopt a statistical metaphor, such methods are not only concerned with the ‘main effects’ on performance of individual system components, but also the shifting, dynamic ‘interactions’ between them.

The authors also outline an evaluation of the methods in terms of ratings of their ability to address a set of theoretical and practical questions (e.g. the degree to which methods capture static/dynamic aspects of tasks and interactions between system levels). The outcomes from the evaluation highlight a set of gaps relating to the coverage and applicability of current methods for sociotechnical systems theory and safety. The final sections of the paper describe a set of future challenges, as well as some practical suggestions for tackling these. These include gaps in theoretical coverage (e.g. coverage of external influences on system performance, the need to delineate system boundaries), as well as practical utility (e.g. support for navigating through and combining the various sociotechnical systems methods in existence, the importance of studies of the reliability and validity of the methods).

## 4. Modelling and simulation of sociotechnical systems and safety

In their paper, Hettinger et al. (2015) discuss issues involved in the development and use of computer-based models and simulations for envisioning and studying workplace safety-related issues in sociotechnical systems. Given the inherent complexity of many such systems, it can often be difficult for those involved in their design, deployment and management to accurately comprehend their structural and dynamic characteristics – particularly the latter. Similarly, empirical studies of sociotechnical systems ‘in the wild’ are severely constrained by researchers' limited ability to introduce structural and/or dynamic experimental manipulations to test hypotheses concerning their impact on system performance and safety-related outcomes. In particular, obvious ethical and logistical constraints generally preclude the ability to conduct field research on the impact to system performance of significant sociotechnical modifications. Clearly one could not, for instance, conduct a field experiment to assess changes in the safe operation of an oil refinery as a function of a controlled series of reductions in staffing combined with a comparable series of increases in production demands. Laboratory-based studies, on the other hand, are generally unable to capture sufficiently the multiple contextual factors that are potentially important influences on the behaviour of sociotechnical systems.

Beyond the obvious ethical and logistical limitations that constrain the ability of traditional empirical techniques to support the scientific study of sociotechnical systems, Hettinger et al. note that there are other compelling features of such systems that argue for a greater emphasis on computer-based modelling and simulation approaches. For instance, any attempt to account for systemic determinants of critical outcomes such as safety (or efficiency, profitability, sustainability, etc.) must be able to cope with the fundamental complexity of sociotechnical systems. Identifying critical system components and achieving a detailed understanding of the causal and feedback relationships that exist between them (and how those relationships vary as a function of context, time, etc.) are both necessary, but not sufficient to support effective empirical examinations. The ability to systematically vary these properties is critical to the formulation of causal inferences about their impact on system outcomes, a task that is perhaps orders of magnitude beyond the scope of even the most sophisticated traditional experimental techniques.

While not offered as a panacea for these difficulties, and bearing in mind the prudent admonition that ‘all models are wrong, but some are useful’ (Box and Draper 1987, 424), Hettinger et al. assert that modelling and simulation afford a potentially useful means for addressing many of the limitations of more traditional empirical methods, particularly in the study of highly complex sociotechnical systems. Similarly, within the domain of system design, deployment and management, appropriately designed models and simulations can support our otherwise limited human capacity to accurately comprehend the performance (past, present or future) of existing or planned sociotechnical systems, particularly as they continue to evolve towards greater complexity.

Several broad classes of computer-based modelling and simulation techniques are delineated with an emphasis on their capabilities and limitations in addressing the issues described previously in the text. These approaches include discrete event modelling, system dynamics modelling, agent-based modelling and various related hybrid approaches. The advantages and disadvantages of each are discussed in detail, along with several representative examples of their application to issues involved in sociotechnical system design and function. To date, there has been little effort to explore the application of such models to problems of workplace safety, although as their use becomes more widespread it is likely that this will change.

Hettinger et al. conclude with a discussion of potential sociotechnical issues that may be particularly amenable to examination within a modelling and simulation framework, both from a specific system design and broader scientific perspective. The analysis of strategies to achieve joint optimisation (or to avoid its antithesis – asynchronous evolution) between technical-work process factors and social-organisational factors is one of several compelling problem areas for which modelling approaches appear to be very well suited. Finally, a critical task that is fundamental to all modelling and simulation approaches is the accurate and reliable identification of any work system's key sociotechnical components, as well as the dynamic communication and control relationships that exist between them.

## 5. Communications and decision-making within sociotechnical systems

In their paper, Flach et al. (2015) address the critical role of communications and decision-making in sociotechnical systems, particularly with respect to their impact on shaping the nature of emergent system properties such as safety. Adopting a broad, dynamical systems framework, the authors explore the ramifications of such a perspective by means of an analysis of two very different work environments; an easily recognisable high hazard installation (a nuclear power plant) and a setting where the sociotechnical complexity may be less obvious (a limited-service, ‘fast food’ restaurant). Specifically, the authors frame their analyses within the conceptual context of a dynamic system comprising multiple, nested sub-systems. Communications are conceived as being related to the construct of ‘observability’ (i.e. how components integrate information to assess the state with respect to local and global constraints). Decisions are related to the construct of ‘controllability’ (i.e. how component sub-systems act to meet local and global safety goals). The safety dynamics of the two sociotechnical systems chosen for analysis are subsequently evaluated as a function of the coupling between observability and controllability across multiple closed-loop components.

In their discussion, Flach et al. invoke one of several overriding sociotechnical concepts, i.e. ‘context matters’. In particular, the authors go to substantial lengths to establish the importance of context, as determined by the influences of entities (and their associated processes) located outside the immediate boundaries of the local work system, in shaping the activity of sociotechnical work systems. Drawing from the work of Rasmussen et al. (1994) and Leveson (2012), the authors discuss the importance of understanding the tight coupling between the local sociotechnical work system and the larger ‘social context’ provided (or, in some cases, imposed) by regulatory, political and cultural ‘ecologies’.

By selecting two case studies that effectively span the exceedingly broad range of sociotechnical complexity within which work systems can potentially exist, Flach et al. address an important practical and theoretical concern relating to the ‘scalability’ of system-based approaches. As noted by Waterson et al. (2015), the majority of contemporary sociotechnical-based approaches to safety have been derived from a concern with the design and operation of highly complex sociotechnical systems (e.g. nuclear power, aviation, petro-chemical processing). In such cases, the many linkages and dependencies between organisational and technical components, while not always readily identifiable, are at least generally recognised as being present and potentially important in the unfolding of various safety-critical situations (see Leveson 2012, for a useful discussion). It is not difficult to conceive of such systems as including multiple, nested sub-systems. However, Flach et al. demonstrate how even where the sociotechnical complexity in particular work systems may not be so readily apparent, they can still be usefully modelled and described as dynamic systems that (a) also comprise multiple nested sub-systems, disruptions of communication and control within any of which can lead to problems with safety, and (b) are directly or indirectly impacted by contextual factors such as legislation, regulations, market forces and others.

Flach et al. acknowledge that their approach offers no simple prescriptions for achieving safety in sociotechnical systems. However, their analyses provide a compelling logic that suggests that thoughtful examination of communication (control) and decision-making (observability) relationships between system components, including those seemingly remote (physically and/or organisationally) from the local work system, can afford useful insights for identifying hazards before accidents occur, or for understanding potential causal factors when they do.

## 6. Sociotechnical attributes of safe and unsafe systems

In the final paper of this special issue, Kleiner et al. (2015) integrate many of the themes from the preceding papers into a discussion of potential sociotechnical attributes of safe and unsafe work systems. Recognising again that there are no *permanently* safe or unsafe systems, the authors focus on the identification of sociotechnical attributes that are potentially useful in identifying the current and projected status of work systems with respect to risks of accidents and worker injury.

The authors select three broad perspectives on system design and operation as vehicles for their discussion. These include human-systems integration (HSI, a domain generally referred to as ‘human factors integration’ in Europe) (Booher 2003), macroergonomics and safety climate (e.g. Huang et al. 2013; Zohar 2010, 2014; Murphy, Robertson, and Carayon 2014). Each of these perspectives adopts a sociotechnical framework within which several consistent features of relatively safe and unsafe systems can be derived. HSI, having originated as a systems design discipline primarily derived from systems engineering, approaches workplace safety from a human-centred or ‘use-centred’ (Flach and Dominguez 1995) framework in which safe and effective human-systems behaviour represents the principal criteria against which design is assessed. From the HSI perspective, systems and organisations that incorporate user input at all stages of system design, deployment, operation and maintenance are in a better position to anticipate risk and effectively address than those who do not. Although HSI has largely focused on issues associated with system design, personnel selection and training, and other human-centred aspects of complex systems, macroergonomics has focused on the role played by the many organisational and cultural factors outside of the immediate work setting that impact safe and effective work performance. These include the impact of organisational policy, communications and decision-making in the design and use of work systems and processes. Although HSI and macroergonomics place approximately equal emphasis on the importance of use-centred design, macroergonomics adopts a more holistic perspective on overall work system design and use by taking into account the organisational and cultural impacts on work systems that HSI has historically under-emphasised. Finally, safety climate focuses on the critical importance of consistency between the safety *policies* espoused by organisations and the actual *practice* of safety within the organisation as evidenced by rewards, punishments, amount and quality of resources dedicated to safety, etc. A domain derived primarily from industrial and organisational psychology, safety climate has experienced a rapid growth in interest in recent years. Having focused its work primarily on organisational determinants of safety climate, the authors note that future work should also examine its more technology-focused aspects and external environmental factors.

Hettinger et al. contrast the overlapping perspectives of HSI, macroergonomics and safety climate with more traditional approaches to safety, focusing their critique on traditional human factors and ergonomics (HFE) and the primarily engineering-centric approach of ‘system safety’ (e.g. O'Keefe 2002). Macroergonomics, having been conceived in reaction to a perceived over-reliance on reductionist perspectives within HFE, has been a significant driver of sociotechnical thinking. The authors argue that of all contemporary HFE schools of thought, macroergonomics perhaps best captures the holistic intent of the sociotechnical school of thought. HSI, on the other hand, arose largely within operational, system design environments (primarily related to complex US Department of Defense programs) that gave rise to system safety. However, whereas system safety largely envisions safety as a function of system hardware and software engineering requirements and characteristics, although more recent iterations of this approach are beginning to incorporate HSI principles (e.g. MIL-STD-882E 2012), HSI adopts a more integral framework within which human interactions with the engineering components of work systems are considered to comprise areas of high safety risk. This helps to illuminate the importance of examining human-in-the-loop aspects of engineering system design. Finally, safety climate, in contrast to traditional HFE, system safety and (to a large extent) HSI, is continuing to focus attention on the key role of consistency between organisational policy and practice (e.g. Murphy, Robertson, and Carayon 2014).

## 7. Future research directions

The papers in this special issue of *Ergonomics* discuss in detail current theoretical issues and research contributions within the field of sociotechnical systems and safety. Future research on sociotechnical systems for workplace safety will benefit from the extensive body of knowledge on sociotechnical methodologies that is described in the paper on ‘Defining research methodologies’ (Waterson et al. 2015).

In the presentation and discussion of the papers at the *Hopkinton Conference*, several consistent issues and themes emerged that illuminated key areas for future work. These include aspects related to (a) coping with an apparent increasing diversity in sociotechnical frameworks, models and methods, (b) the challenge of understanding causality across the various layers of sociotechnical systems and (c) understanding the nature and impact of system design and performance trade-offs as they impact workplace safety.

The special issue papers provide a number of reviews of sociotechnical models, frameworks and methods. These clearly cover an enormous amount of ground and draw on a tradition of work extending from the 1950s up to the present day. These reviews are in themselves far from exhaustive – given more time and space many more approaches, models and methods could have been surveyed. A survey of the field of accident analysis and investigation for example, would most likely result in a huge array of techniques for modelling and probing deeper into the factors contributing to human error. A range of these techniques, including one of the most recent, systems theoretic accident models and processes (STAMP) (Leveson 2012), are described in the special issue.

The growth of different types of frameworks, models and methods can be seen as evidence of the success of sociotechnical systems theory. Likewise, we might indeed expect to find such a rich diversity, given the widely varying properties and characteristics of different work domains and their associated safety challenges. As a number of the papers emphasise, safety is a dynamic, emergent and constantly changing open system in itself, subject to a wide range of technological and other types of environmental influences. The likelihood that new models will be needed in the future is very high (Le Coze 2013). Furthermore, we also need research that addresses the design of sociotechnical systems for different external environments, in particular for different cultural groups. Consideration of national culture values, attitudes, behaviour patterns and customs need to be systematically considered and incorporated into the design of organisational structure and systems, along with the technology sub-system (Scott 2008; Meshkati and Robertson 1986). Organisations need to accommodate for and be more inclusive of the ever changing workforce cultural diversity by considering different structural changes to the work system, the life cycle of the technology and knowledge transfer (Shahnavvz 2002), as well as the impact these factors may have on safety culture and climate (Reader et al. in press). What are the invariant characteristics of sociotechnical systems for workplace safety across cultural groups? What are the characteristics of sociotechnical systems that need to be adapted for different cultural groups in order to improve workplace safety? This type of research could also be expanded to examine other aspects of the external environment, such as safety and industry standards.

It also seems likely that there will be a need to consolidate and, in some cases, integrate current models, frameworks and methods (Hovden, Albrechtsen, and Herrera 2010), particularly where some of these derive from specific disciplines and fields of expertise (e.g. models of occupational safety and health; e.g. Burton 2010) compared to those available in complex systems safety (e.g. Leveson 2013) and healthcare (e.g. Carayon et al. 2006). A related issue raised at the *Hopkinton Conference* concerned the need to improve the reliability and validity of sociotechnical methods and tools (see also Stanton 2013).

Understanding causality across system levels was discussed as a critical challenge for understanding risks to safety, particularly in highly complex sociotechnical systems. Sociotechnical systems theory concerns itself with the design of entire work systems, and models have been proposed to help guide researchers in identifying salient job and organisational level variables, which influence safety and safe behaviour. However, few theories or models explicitly provide insight into causal pathways and mechanisms that operate between levels of the work system. As a partial solution, Karsh (2006) proposed the term ‘mesoergonomics’ in order to argue for an open systems approach to ergonomics theory and research ‘whereby the relationship between variables in at least two different levels or echelons is studied, where the dependent variables are human factors and ergonomic constructs’ (Karsh, Waterson, and Holden 2014, 2). Attempts to examine interactions between ‘micro’ and ‘macro’ levels of analysis are not a recent concern (see for example, Wilson and Grey 1990); however, it continues to be an important goal if we are to understand and improve workplace safety.

The need to understand and provide better means of quantifying the various trade-offs that impact safety (e.g. reliability, quality, costs) was also raised as a critical issue for sociotechnical systems approaches to safety (see also Wilson et al. 2009). This was seen as particularly important given the current climate of economic austerity and associated frequent reductions in worker numbers, assuming there to be human and technological redundancy, but resulting in possible erosion of system resilience. Additional trade-offs include psychological phenomena such as the balance between safety compliance and rule breaking (Bieder and Bourrier 2013; Hale and Borys 2013a, 2013b); the drive to achieve ‘resilience’ in complex systems (Hollnagel 2006) and the interplay between reliability, validity and utility in the development of HFE methods (Waterson, Clegg, and Robinson2014).

Given that workplace safety is one of many goals that organisations need to achieve, future research should explore how a sociotechnical systems approach may help to balance different organisational objectives, along with the competing demands. In considering safety climate as a measure of joint optimisation, how we can operationalise and measure join optimisation, for example. As such, it is difficult to view safety climate measures as diagnostic or audit-type tools. A highly positive overall safety climate score may, indeed, reflect a high degree of social-technical optimisation, but a low score does not reveal the specific nature of any deficiencies or what needs to be done to improve the situation (Murphy, Robertson, and Carayon 2014).

The sociotechnical systems model for workplace safety could help in bridging the gap between the science and practice of workplace safety. The model could be used by safety practitioners to understand the role of the broader socio-organisational context in fostering safety and to devise more powerful, sustainable solutions for enhancing safety. Researchers could use the expertise of safety practitioners to describe the sociotechnical system characteristics of importance for workplace safety. This research would, for instance, expand the research conducted by National Institute of Occupational Safety and Health (NIOSH) researchers in the 1970's (Cohen, Smith, and Cohen 1975) on characteristics of companies that promote or hinder workplace safety.

Research on sociotechnical systems for workplace safety should also assess the impact of interventions on workplace safety. As we accumulate intervention research studies, similarities and differences in the content and process characteristics of effective sociotechnical interventions will emerge, such as incorporating a participatory ergonomics approach (Robertson et al. 2013), and this information will need to be disseminated to safety practitioners for their adoption.

## 8. Conclusion

The papers comprising this special issue provide many insights into critical issues, themes and research opportunities within a sociotechnical systems perspective on workplace safety. In light of the many decades that have passed since the original insights of the Tavistock Institute (e.g. Trist and Bamforth 1951), it is perhaps surprising that with several notable exceptions (as discussed by Waterson et al. 2015) this area has remained relatively under-developed until much more recently. Nevertheless, a clear consensus is emerging, both among those scientists who participated in the *Hopkinton Conference* as well as others (e.g. Haslam et al. 2005; Coakes and Coakes 2009; Hollnagel 2009; Wilson 2014), that safety improvements in increasingly complex work environments will substantially benefit from an approach that focuses on the dynamic network of safety-critical interactions between organisational and technical system components. Simply put, the increasing complexity of contemporary work systems has created the demand for new research, analysis and design methods that acknowledge and adequately account for the dynamic, complex and adaptive nature of such systems.

Sociotechnical systems theory is no longer in its infancy. Macroergonomics, HSI, system ergonomics, cognitive systems engineering, safety climate research, resilience engineering and the STAMP approach (Leveson 2012) are all firmly based in a sociotechnical world view. The challenge that remains, as discussed in the articles included in this special issue of *Ergonomics,* is the application of sociotechnical principles to the amelioration of the significant and unacceptable toll, in human and economic terms, of accidents and injuries in the workplace.

## References

[cit0001] Bieder C., Bourrier M. (2013). Trapping Safety into Rules.

[cit0002] Booher H. R. (2003). Handbook of Human-Systems Integration.

[cit0003] Box G. E. P., Draper N. R. (1987). Empirical Model Building and Response Surfaces.

[cit0004] Burton J. (2010). WHO Healthy Workplace Framework and Model: Background and Supporting Literature and Practices. http://www.who.int/occupational_health/healthy_workplace_framework.pdf.

[cit0005] Carayon P., Hancock P., Leveson N., Noy Y. I., Sznelwar L., van Hootegem G. (2015). Advancing a Sociotechnical Systems Approach to Workplace Safety: Developing the Conceptual Framework.

[cit0006] Carayon P., Schoofs Hundt A., Karsh B. T., Gurses A. P., Alvarado C. J., Smith M., Flatley Brennan P. (2006). Work System Design for Patient Safety: The SEIPS Model. Quality and Safety in Health Care.

[cit0055] Clegg C. W. (2000). Sociotechnical Principles for System Design. Applied Ergonomics.

[cit0007] Coakes E., Coakes J. (2009). A Meta-Analysis of the Direction and State of Sociotechnical Research in a Range of Disciplines. International Journal of Sociotechnology and Knowledge Development.

[cit0008] Cohen A., Smith M. J., Cohen H. H. (1975). Safety Program Practices in High vs. Low Accident Rate Companies: An Interim Report (Questionnaire Phase).

[cit0009] Dainoff M. J. (2009). Can't We All Just Get Along? Some Alternative Views of the Knowledge Worker in Complex HCI Systems. International Journal of Human-Computer Interaction.

[cit0056] Dekker S. (2011). Drift into Failure.

[cit0010] Dekker S. (2015). Safety Differently.

[cit0012] Flach J. M., Carroll J. S., Dainoff M. J., Hamilton W. I. (2015). Striving for Safety: Communicating and Deciding in Sociotechnical Systems.

[cit0011] Flach J. M., Dominguez C. O. (1995). Use-Centered Design: Integrating the User, Instrument, and Goal. Ergonomics in Design.

[cit0014] Hale A., Borys D. (2013a). Working to Rule, or Working Safely? Part 1: A State of the Art Review. Safety Science.

[cit0015] Hale A., Borys D. (2013b). Working to Rule or Working Safely? Part 2: The Management of Safety Rules and Procedures. Safety Science.

[cit0013] Hale A. R., Hovden J., Feyer A. M., Williamson A. (1998). Management and Culture: The Third Age of Safety: A Review of Approaches to Organisational Aspects of Safety, Health and Environment.

[cit0016] Haslam R. A., Hide S. A., Gibb A. G. F., Gyi D. E., Pavitt T., Atkinson S., Duff A. R. (2005). Contributing Factors in Construction Accidents. Applied Ergonomics.

[cit0017] Hendrick H. W., Kleiner B. M. (2002). Macroergonomics: Theory, Methods, and Applications.

[cit0018] Hettinger L. J., Kirlik A., Goh Y. M., Buckle P. (2015). Modeling and Simulation of Complex Sociotechnical Systems: Envisioning and Analyzing Work Environments.

[cit0019] Holland J. H. (2012). Signals and Boundaries: Building Blocks for Complex Adaptive Systems.

[cit0057] Hollnagel E., Hollnagel E., Woods D., Leveson N. (2006). Resilience—the Challenge of the Unstable.

[cit0020] Hollnagel E. (2009). The ETTO Principle: Efficiency-Thoroughness Trade-Off: Why Things that Go Right Sometimes Go Wrong.

[cit0021] Hovden J., Albrechtsen E., Herrera I. A. (2010). Is there a Need for New Theories, Models and Approaches to Occupational Accident Prevention?. Safety Science.

[cit0022] Huang Y., Zohar D., Robertson M., Garabet A., Lee J., Murphy L. (2013). Development and Validation of Safety Climate Scales for Lone Workers Using Truck Drivers as Exemplar. Transportation Research Part F: Traffic Psychology and Behaviour.

[cit0023] Karsh B. (2006). Meso-Ergonomics: A New Paradigm for Macroergonomics Research.

[cit0024] Karsh B.-T., Waterson P. E., Holden R. J. (2014). Crossing Levels in Systems Ergonomics: A Framework to Support ‘Mesoergonomic’ Inquiry. Applied Ergonomics.

[cit0025] Kasvi J. J., Vartiainen M., Pulkkis A., Nieminen M. (2000). The Role of Information Support Systems in the Joint Optimization of Work Systems. Human Factors and Ergonomics in Manufacturing & Service Industries.

[cit0026] Kelly J. E. (1978). A Reappraisal of Sociotechnical Systems Theory. Human Relations.

[cit0027] Klein L. (2014). What Do We Actually Mean by ‘Sociotechnical’? On Values, Boundaries and the Problems of Language. Applied Ergonomics.

[cit0028] Kleiner B. M., Hettinger L. J., DeJoy D. M., Huang Y. H., Love P. E. D. (2015). Sociotechnical Attributes of Safe and Unsafe Systems.

[cit0029] Le Coze J. -C. (2013). New Models for New Times: An Anti-Dualist Move. Safety Science.

[cit0030] Leveson N. (2012). Engineering a Safer World.

[cit0031] Leveson N. (2013). Perspective: The Drawbacks in Using the Term ‘System of Systems’. Biomedical Instrumentation and Technology.

[cit0058] Meshkati N., Robertson M. M., Hendrick H. W., Brown O. (1986). The Effects of Human Factors on the Success of Technology Transfer Projects to Industrial Developing Countries: A Review of Representative Case Studies.

[cit0032] MIL-STD-882E (2012). US Department of Defense Standard Practice: System Safety. http://www.system-safety.org/documents/mil-std-882e.pdf.

[cit0033] Miller J. H., Page S. E. (2007). Complex Adaptive Systems: An Introduction to Computational Models of Social Life.

[cit0034] Mumford E. (2006). The Story of Socio-Technical Design: Reflections on its Successes, Failures and Potential. Information Systems Journal.

[cit0035] Murphy L. A., Robertson M. M., Carayon P. (2014). The Next Generation of Macroergonomics: Integrating Safety Climate. Accident Analysis and Prevention.

[cit0036] Noy Y. I., Hettinger L., Dainoff M., Carayon P., Leveson G., Courtney T., Robertson M. (2015). Emerging Issues in Sociotechnical Systems Thinking and Workplace Safety.

[cit0037] O'Keefe D. (2002). MIL-STD-882 – Its History; and Importance. In System Safety: A Science and Technology Primer. http://www.system-safety.org/resources/ss_primer_4_02.pdf.

[cit0059] Rasmussen J., Pejtersen A. M., Goodstein L. P. (1994). Cognitive Systems Engineering.

[cit0038] Reader T. W., Noort M. C., Shorrock S., Kirwan B. (in press). Safety San Frontières: An international Safety Culture Model. Risk Analysis.

[cit0039] Reiman T., Rollenhagen C., Pietikäinen E., Heikkilä J. (2015). Principles of Adaptive Management in Complex Safety–Critical Organizations. Safety Science.

[cit0040] Robertson M. M., Courtney T. K. (2004). A Systems Analysis Approach to Solving Office Work System Health and Performance Problems. Theoretical Issues in Ergonomics Science.

[cit0042] Robertson M. M., Henning R., Warren N., Nobrega S., Dove-Steinkamp M., Tibirica L., Bizarro A., the CPH-NEW Research Team (2013). The Intervention Design and Analysis Scorecard. Journal of Occupational Environmental Medicine.

[cit0041] Robertson M. M., Kleiner B., O'Neill M. J., Hendrick H., Kleiner B. (2002). Macroergonomics Sytems Tools.

[cit0060] Scott P. A., Zink K. J. (2008). The Role of Ergonomics in Securing Sustainability in Dveloping Cuntries.

[cit0061] Shahnavaz H., Hendrick H. W., Kleiner B. M. (2002). Macroergonomic Considerations in Technology Transfer.

[cit0043] Stanton N. A. (in press). Commentary on the Paper by Heimrich Kanis Entitled ‘Reliability and Validity of Findings in Ergonomics Research’: Where is the Methodology in Ergonomics Methods?. Theoretical Issues in Ergonomics Science.

[cit0044] Stanton N. A., Baber C., Harris D. (2008). Modelling Command and Control: Event Analysis of Systemic Teamwork.

[cit0045] Trist E., Bamforth K. (1951). Some Social and Psychological Consequences of the Longxwall Method of Coal-Getting: An Examination of the Psychological Situation and Defences of a Work Group in Relation to the Social Structure and Technological Content of the Work System. Human Relations.

[cit0046] Underwood P., Waterson P. (2014). Systems Thinking, the Swiss Cheese Model and Accident Analysis: A Comparative Systemic Analysis of the Grayrigg Train Derailment Using the ATSB, AcciMap and STAMP Models. Accident Analysis & Prevention.

[cit0047] von Bertalanffy L. (1968). General Systems Theory: Foundations, Development and Applications.

[cit0049] Waterson P. E., Clegg C. W., Robinson M., Broberg O., Fallentin N., Hasle P., Jensen P. L., Kabel A., Larsen M. E., Weller T. (2014). Trade-Offs Between Reliability, Validity and Utility in the Development of Human Factors Methods.

[cit0048] Waterson P., Robertson M. M., Cooke N. J., Militello L., Roth E., Stanton N. A. (2015). Defining the Methodological Challenges and Opportunities for an Effective Science of Sociotechnical Systems and Safety.

[cit0050] Wilson J. R. (2014). Fundamentals of Systems Ergonomics/Human Factors. Applied Ergonomics.

[cit0051] Wilson J. R., Grey S. M., Haslegrave C. M., Wilson J. R., Corlett E. N., Manenica I. (1990). But What are the Issues in Work Design?.

[cit0052] Wilson John, Ryan Brendan, Schock Alex, Ferreira Pedro, Smith Stuart, Pitsopoulos Julia (2009). Understanding Safety and Production Risks in Rail Engineering Planning and Protection. Ergonomics.

[cit0053] Zohar D. (2010). Thirty Years of Safety Climate Research: Reflections and Future Directions. Accident Analysis and Prevention.

[cit0054] Zohar D., Schneider B., Barbera B. (2014). Safety Climate: Conceptualization, Measurement and Improvement.

